# Ameliorative effects of high intensity interval training and *Lactobacillus rhamnosus GG Protect* against tetracycline-induced fatty liver in rats: a gene expression profiling comparative study

**DOI:** 10.17179/excli2022-4791

**Published:** 2022-07-21

**Authors:** Hamideh Mahmoodzadeh Hosseini, Hossein Shirvani, Fariba Aghaei, Ehsan Arabzadeh, Martin Hofmeister

**Affiliations:** 1 Applied Microbiology Research Center, Systems Biology and Poisonings Institute, Baqiyatallah University of Medical Sciences, Tehran, Iran; 2 Exercise Physiology Research Center, Life Style Institute, Baqiyatallah University of Medical Sciences, Tehran, Iran; 3 Faculty of Physical Education and Sport Sciences, Karaj Branch, Islamic Azad University, Alborz, Iran; 4 Department of Food and Nutrition, Consumer Centre of the German Federal State of Bavaria, Munich, Germany

**Keywords:** exercise training, Lactobacillus rhamnosus GG, fatty liver disease, probiotic

## Abstract

Exercise training and probiotics have been suggested as a treatment for the prevention of chronic liver damage such as non-alcoholic fatty liver disease (NAFLD). *Lactobacillus rhamnosus Gorbach‐Goldin (LGG)* is one of the most widely used probiotic strains that decreases liver damage. Thus, this study aims to consider the ameliorative effects of high intensity interval training (HIIT) and *LGG* against tetracycline-induced fatty liver in rats. Eighty male Wistar rats were randomly divided into 8 groups of (n=10 each group): control, LGG, HIIT, LGG+HIIT, NAFLD, NAFLD+LGG, NAFLD+HIIT, and NAFLD+LGG+HIIT. The rats are treated by intraperitoneal injection with 140 mg/kg^−1^ tetracycline, an antibiotic previously known to induce steatosis. The exercise training groups performed HIIT 5 days/week for 5 weeks. 10^7^ colony-forming units (cfu) of LGG were gavaged for LGG groups 5 days/week for 5 weeks. Probiotic supplementation in combination with interval training significantly decreased *tissue inhibitor of matrix metalloproteinases-1 (TIMP-1)* mRNA and *matrix metalloproteinase-2* *(MMP-2)* mRNA in the liver (p<0.05), while the levels of *lysosomal acid lipase* *(LIPA)* mRNA was significantly increased compared to NAFLD group. Also, compared with NAFLD group, NAFLD+LGG, NAFLD+HIIT and NAFLD+LGG+HIIT groups showed a significant decrease in hepatic *monocyte chemoattractant protein-1* *(MCP-1)*. Compared to LGG and LGG+HIIT groups, all NAFLD groups showed a significant decrease in *apolipoprotein C3 (apoc3)* in liver tissue (p<0.05). The results suggested that interval exercise with LGG supplementation minimizes cell destruction and inflammation in liver tissue due to NAFLD by improving gene expression profiles.

## Introduction

The liver is a vital organ in maintaining glucose homeostasis whose dysfunction with the wrong lifestyle leads to the induction of various inflammatory and metabolic diseases (Mantovani et al., 2018[35]). The prevalence of various types of liver disease is increasing worldwide. Non-alcoholic fatty liver disease (NAFLD) is a metabolic disorder that includes steatosis, non-alcoholic steatohepatitis (NASH), cirrhosis, and hepatocellular carcinoma. Liver fibrosis is a major determinant of liver-related complications in patients with NAFLD. Some patients with simple steatosis develop liver fibrosis, which can lead to cirrhosis, liver cell carcinoma, liver transplantation, and death. The presence of liver fibrosis is the main determinant of liver complications (Angulo et al., 2015[4]). Previous studies also suggested that steatosis induced by rifampicin, tetracycline, and valproic acid could be, at least in part, increase fatty acid translocase/cluster of differentiation 36 (FAT/ CD36) expression or its activity (Choi et al., 2015[11]). Among the factors that indicate the prevalence and spread of liver fibrosis is the tissue inhibitor of matrix metalloproteinases-1 (TIMP-1).

It has been shown that serum TIMP-1 levels increase in patients with chronic liver disease, which is associated with the histological grade of liver fibrosis. Iredale et al. found that TIMP-1 mRNA was increased in liver damages (Iredale et al., 1995[23]). mRNA expression and protein content of TIMP-1 and TIMP-2 were significantly increased in advanced fibrotic liver. TIMP-1, as a member of the TIMP family, is a major inhibitor of matrix metalloproteinases (MMPs) and therefore is directly involved in the regulation and regeneration of extracellular matrix (ECM) in all human organs (Gressner and Weiskirchen, 2006[20]). MMPs are zinc-dependent endopeptidases that are involved in the degradation of extracellular proteoglycans and the breakdown of matrix proteins (Maquoi et al., 2003[36]). MMP-2 is a type IV collagenase that is secreted as a proenzyme from a variety of cells, including liver satellite cells, and can modulate the extracellular environment. Increased expression and increased MMP-2 levels have been observed in diabetic rats with liver cirrhosis (Salama et al., 2013[47]). MMP-2 also plays an important role in hepatic vascular homeostasis. This factor also mediates the activation of fibrogenesis markers of transforming growth factor beta (TGF-β). MMP-2 can modulate the activation of inflammatory markers, including tumor necrosis factor-alpha (TNF-α) (Duarte et al., 2015[18]). TNF-α is a proinflammatory cytokine and plays an important role in the development of the non-alcoholic fatty liver. Monocyte chemoattractant protein-1 (MCP-1) is meanwhile a chemokine responsible for the migration and uptake of macrophages and monocytes into the liver following inflammation (Deshmane et al., 2009[17]). Preclinical studies show that MCP-1 does not contribute to steatosis or inflammation but independently increases fibrosis. However, few studies have examined the role of MCP-1 in NAFLD. Alpha-smooth muscle actin (α-SMA) is also often used as a marker of myofibroblast formation and plays an important role in fibrogenesis (Carpino et al., 2005[9]). Myofibroblasts are metabolically and morphologically distinct fibroblasts that express α-SMA, and their activation plays a major role in the fibrotic response. Therefore, a study of α-SMA activity in fatty liver fibrosis can also have a therapeutic purpose. Lysosomal acid lipase (LIPA) is another factor in hepatic fibrosis because LIPA deficiency has been shown to lead to progressive microvascular hepatostasis, fibrosis, cirrhosis, dyslipidemia, and vascular disease (Baratta et al., 2019[5]). Also, patatin-like phospholipase domain-containing 3 (PNPLA3) is mainly expressed in the liver and it is believed that defects and mutations in PNPLA3 may be associated with hepatic insulin resistance and hepatic fibrosis (Fan et al., 2016[19]). It seems that controlling the expression of these genes associated with liver fibrosis can also have a therapeutic aspect.

Because the pathogenesis of NAFLD is not fully understood, recent studies have focused on identifying NAFLD-related genes, particularly those candida genes involved in fat metabolism, insulin regulation, and obesity (Dabravolski et al. 2021[16]). A number of previous studies have examined the genetic impact of apolipoprotein C3 (APOC3) polymorphisms on NAFLD (Jain et al., 2019[24]; Xu et al. 2020[56]). APOC3 is synthesized in the liver and small amounts in the intestine and is found in triglyceride-rich lipoproteins and high-density lipoproteins. Transgenic mice that overexpress human APOC3 have been shown to be more susceptible to NAFLD and insulin resistance (Lee et al., 2011[29]). Another factor affecting fat metabolism is acetyl-CoA carboxylase (ACC), which has also emerged as a therapeutic target for NAFLD. Mice with ACC elimination have been observed to have higher levels of fatty acid oxidation in skeletal muscle and heart, which gives protection against dietary obesity and hepatic steatosis (Abu-Elheiga et al., 2003[2]).

Based on the evidence, the use of anti-inflammatory interventions can be effective in the treatment of metabolic diseases. Regular exercise can reduce inflammation, fibrosis, and liver damage by suppressing macrophages (Kawanishi et al., 2012[27]). Recent studies on the relationship between exercise and the liver have shown that exercise has a significant effect on reducing liver fat (Johnson et al., 2012[25]; van der Windt et al., 2018[50]; Cigrovski Berkovic et al., 2021[12]). In addition to exercise, it is stated that probiotic therapy is also effective in reducing liver damage (Castillo et al., 2021[10]). Ritze et al. (2014[44]) observed that *Lactobacillus rhamnosus Gorbach‐Goldin (LGG)* protects against non-alcoholic fatty liver disease in mice (Ritze et al., 2014[44]). Wang et al. also showed that *Bifidobacterium adolescentis* and LGG alleviate NAFLD induced by a high-fat, high-cholesterol diet through modulation of different gut microbiota-dependent pathways (Wang et al., 2020[52]). However, hitherto, no study has investigated the simultaneous effect of exercise, especially HIIT with LGG, on the profile of some destructive genes in NAFLD. Therefore, the purpose of the present study was to consider the ameliorative effects of interval exercise and *Lactobacillus rhamnosus GG *against tetracycline-induced fatty liver in rats.

## Materials and Methods

### Animals

**Figure 1:**
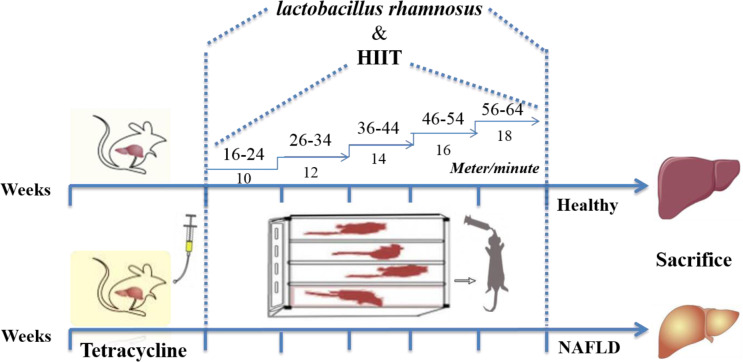
A protocol schematic detailing the timeline of all gavage and training visits

Eighty, male Wistar rats (av. weight of about 220 g) were obtained from the Pasteur Institute of Iran, Tehran, Iran. Animals were acclimated to the novel environment for 1-2 weeks. They were placed alone in metal cages at controlled room temperature, and their living conditions were considered as a 12-12-hour light-dark cycle with a humidity of around 50 %. The animals were then randomly divided into the following eight experimental groups: (i) Group 1: control (received drinking water), (ii) Group 2: LGG (received *L. rhamnosus *GG) (iii) Group 3: HIIT (performed HIIT for five weeks), (iv) Group 4: LGG+HIIT (received *L. rhamnosus *GG + performed HIIT for five weeks), (v) Group 5: NAFLD (received tetracycline for 7 days), (vi) Group 6: NAFLD+LGG (received tetracycline + received *L. rhamnosus *GG*),* (vii) Group 7: NAFLD+HIIT (received tetracycline + performed HIIT), (viii) Group 8: NAFLD+LGG+HIIT (received tetracycline + received *L. rhamnosus *GG + performed HIIT (Figure 1(Fig. 1)). All animals were behaved and sacrificed following the National Institutes of Health guide for the care and use of laboratory animals (NIH Publications No. 8023, revised 1978). The experimental procedures were all performed according to the approval of the Ethical Committee of Baqiyatallah University of Medical Sciences, Tehran, Iran (ethical code: IR.BMSU.REC.1396.716). 

### Hepatic steatosis

Hepatic steatosis induction was performed according to the previously developed method (Shabana et al., 2012[48]). Rats of the groups 5 to 8 received intragastrically the suspension of tetracycline hydrochloride (Vitaminy, LTD, Ukraine) dissolved in 2 ml of water daily in a dose of 140 mg/kg of body weight, which was determined by daily weighing, during 7 days. This dose leads to a constant toxic effect of tetracycline hydrochloride on liver tissue during the experiment. Animals in control group received drinking water daily.

### LGG

*L. rhamnosus* GG (PTCC 1637) was cultured in *Lactobacillus *De Man, Rogosa, and Sharpe broth (MRS broth; Difco, BD, Sparks, MD) at 37 °C in accordance with PTCC guidelines. Bacteria were harvested from MRS broth by centrifugation and colony-forming units counted by dilution and streaking on MRS agar plates (Difco) at 37 °C overnight. LGG was then centrifuged and resuspended at a dilution of 2.5 × 10^7^ CFU/ml in PBS and 1 mL gavage was used for once-a-day treatment (after tetracycline hydrochloride addition).

### Exercise training protocol

The training program began by adapting rats to the apparatus for seven days by placing them on a motor-driven treadmill (Iranian Tajhiz Gostar, 2016, Tehran, Iran). The training protocol at the first week began with the rats receiving exercise on the treadmill at 16-24 meters/minute for 2 min and 1-min active rest at 10 meters/min for 5 sets. One week after the initial stage, the time and speed of running were increased so that the intensity of exercise in the last week reached 56-64 meters/min (2 min) and 18 meters/min (1 minute) active rest (Kalaki-Jouybari et al., 2020[26]). This exercise training protocol was performed for 5 weeks 5 days/week (Figure 1(Fig. 1)). The angle of inclination was 0 ° over the whole study period. During 5 weeks, the warm up and cool down consisted of 4-12 meter/min for 5 min.

### Histological analysis

Liver segments were removed and fixed in 4 % buffered formalin. Formalin-fixed livers were embedded in paraffin, sectioned at 5-μm thickness, and stained with hematoxylin and eosin. Histological analysis was evaluated based on the scoring criteria as follows (Merat et al., 2010[37]): steatosis 0: <5 %; 1: 5-33 %; 2: 34-66 %; 3: >66 %.

### Gene expression

Total RNA was isolated utilizing guanidine/phenolsolution (Qiazol-Qiagen USA). RNA quantity and quality were assessed by NanoDrop 2000 (Thermo scientific). One μg of total RNA was taken for the first-strand complementary DNA (cDNA) synthesis reaction (Hyper script RT PCR-GeneAll) according to the manufacturer's protocol. The real-time PCR was performed on a CFX 384 Bio-Rad thermal cycler (Bio-Rad). One microliter of cDNA of each sample was amplified using SensiMiX(Bioline) SYBR Green PCR Master Mix (2X) (Applied Biosystems-Amplicon, catalogue number 4309155) with 7500 Fast Sequence detector (Applied Biosystems, Foster City, CA, USA). The housekeeping gene GAPDH was measured in parallel as an internal control. The thermal profile used for the qRT‐PCR had three stages: 95 °C for 3 min (1 cycle); 95 °C, 57 °C, and 72 °C for 30 seconds each (40 cycles); 95 °C for 15 seconds and then 60 °C for 1 h (1 cycle). The fold change for each gene was determined after normalization to GAPDH using the 2−ΔΔCT method (Livak and Schmittgen, 2001[34]). 

ΔCT= CT_target _-Ct_reference_

ΔΔCt= ΔCT_test sample_-ΔCT_control sample_

Relative expression:2^- ΔΔCt^

The results are represented as the mean (± standard error of mean SEM) fold changes with respect to the sham control. 

Primer sequences used are shown in Table 1(Tab. 1).

### Statistical analysis

All data are expressed as means ± standard error (SE). Differences among groups were determined using a one-way analysis of variance followed by Tukey's post hoc test. Statistical analyses were performed using IBM SPSS Statistics 23.0 (Armonk, NY: IBM Corp.). P < 0.05 was considered statistically significant.

## Results

### Hepatic steatosis confirmation (H&E)

**Figure 2:**
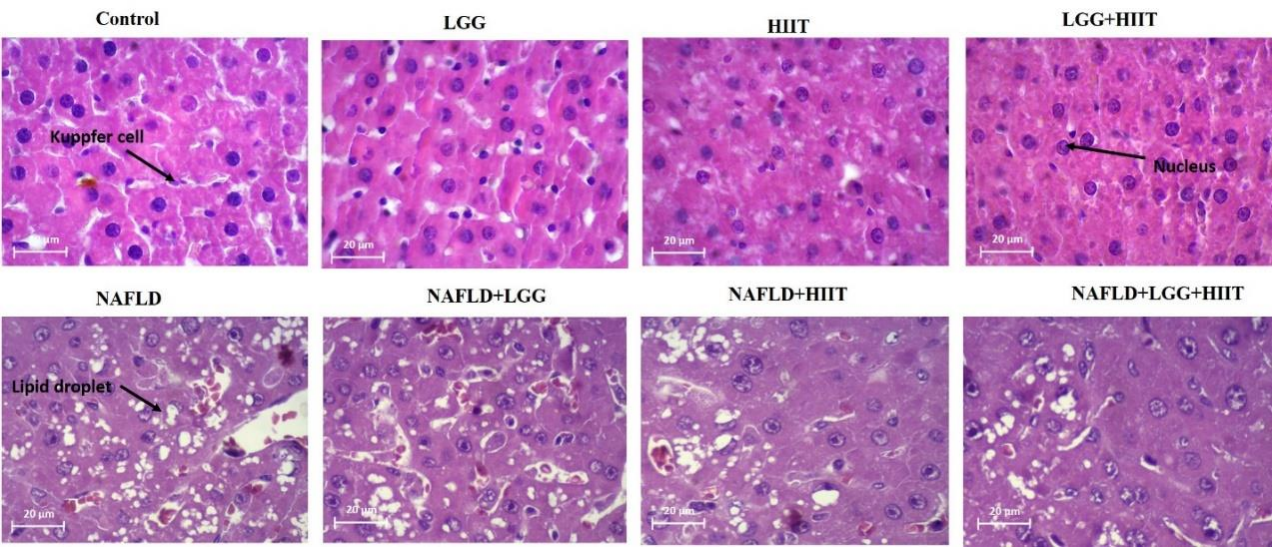
Effects of HIIT on hepatic steatosis. Representative images of hematoxylin and eosin (H&E, magnification 20 um). LGG: Probiotic *L. rhamnosus* GG, HIIT: High intensity interval training, NAFLD: Non-alcoholic fatty liver disease

NAFLD is characterized by excess fat accumulation and steatosis in the liver tissue. We determined hepatic steatosis using hematoxylin and eosin (H&E) staining (Figure 2(Fig. 2)). Based on the results obtained in the healthy control group, it was found that hepatic cells had a normal appearance. In the interlobular space, hepatic triad and central vein with normal appearance were observed. Kupffer cells were found around the sinusoidal vessels. Hepatocytes were identified in small numbers without nuclei and with a similar appearance to apoptotic cells. Normal-shaped liver cells were also found in the probiotic and HIIT groups. Meanwhile, in probiotic and HIIT groups, a hepatic triad was observed around the lobule and in the center of each central venous lobule. The percentage of nucleated hepatocytes with the appearance of apoptosis in these two groups was about 5 %. This tissue appearance was also identified in the combined group of exercise and probiotics, with the difference that the number of apoptotic cells was less than 5 % in this group.

However, the NAFLD group had more lipid droplets and micro- and macrovesicular steatosis than the healthy control group. Also, in NAFLD groups, cells without nuclei or with fragmented nuclei were observed in about 70 % of the tissue. Lymphocyte cell secretion was observed in the intercellular spaces. A hepatic triad was found in the interlobular space and between each lobule of the central vein. Kupffer cells were observed around sinusoidal arteries more those than in healthy controls. In other NAFLD groups, the appearance of tissue showed the death of a number of liver cells; however, this rate was about 40 % in probiotic group, about 30 % in the exercise group, and about 15 % in the combined group of exercise and probiotic. In the end, these results show that 5-week of HIIT and probiotic supplementation in NAFLD group attenuated hepatic steatosis in comparison with Control NAFLD (Figure 2(Fig. 2)). 

### TIMP-1 mRNA

**Figure 3:**
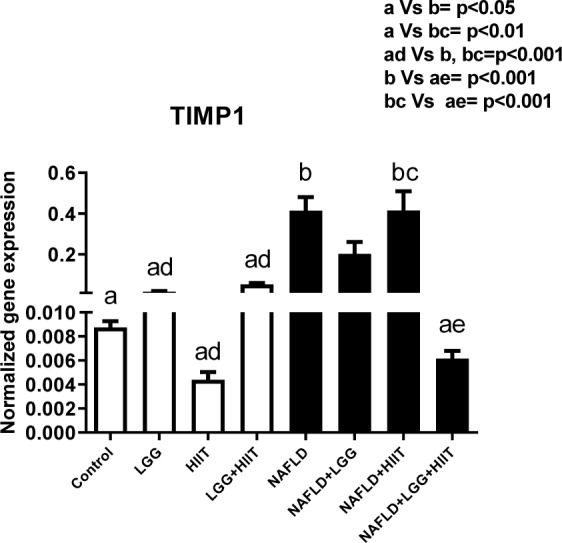
Liver tissue inhibitor matrix metalloproteinase 1 (*timp*1) mRNA expression in different groups of study. All values are the mean ± SD (n = 10 per group). Values with different superscripts are significantly different, p < 0.05. LGG: Probiotic *L. rhamnosus* GG, HIIT: High intensity interval training, NAFLD: Non-alcoholic fatty liver disease

Tissue inhibitor matrix metalloproteinase-1 (*TIMP-1*) was assessed by RT-PCR. *TIMP-1* mRNA was elevated in NAFLD and NAFLD+HIIT rats (p < 0.05 and p < 0.01, respectively) compared to control rats. Additionally, NAFLD and NAFLD+HIIT increased hepatic *TIMP-1* gene expression compared to LGG, HIIT and LGG+HIIT groups (p* < *0.001). In consideration of fatty liver groups, the results showed that the combination therapy group (NAFLD+LGG+ HIIT) caused a significant decrease in *TIMP-1* mRNA compared to the NAFLD group (p < 0.001) (Figure 3(Fig. 3)).

### MMP-2 mRNA

**Figure 4:**
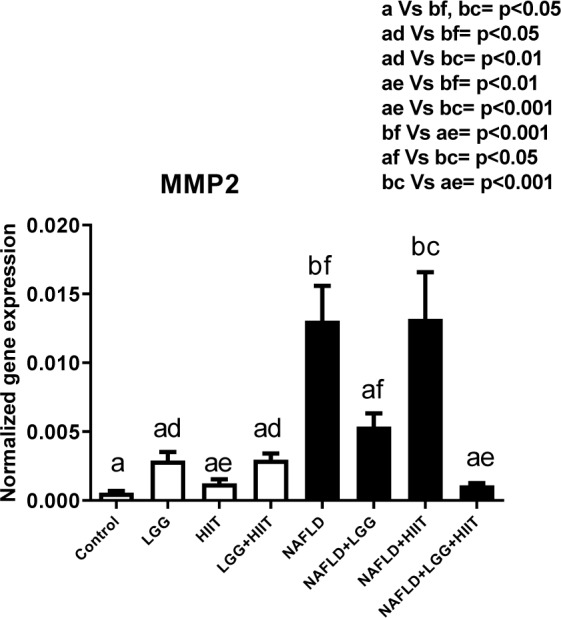
Liver tissue inhibitor matrix metalloproteinase 2 (*mmp2*) mRNA expression in different groups of study. All values are the mean ± SD (n = 10 per group). Values with different superscripts are significantly different, p < 0.05. LGG: Probiotic *L. rhamnosus* GG, HIIT: High intensity interval training, NAFLD: Non-alcoholic fatty liver disease

Changes in gene expression of *MMP-2* are shown in Figure 4(Fig. 4). As can be seen, compared to the healthy control group, only the NAFLD and NAFLD+HIIT groups showed a significant increase in *MMP-2* gene expression (for both p < 0.05). Furthermore, NAFLD and NAFLD+HIIT groups showed a significant increase in liver *MMP-2* compared to healthy probiotics, healthy HIIT and probiotic, and HIIT groups (p < 0.05). Compared to the NAFLD group, the NAFLD+LGG+HIIT group showed a significant decrease in gene expression of *MMP-2* (p < 0.001).

### α-SMA mRNA

**Figure 5:**
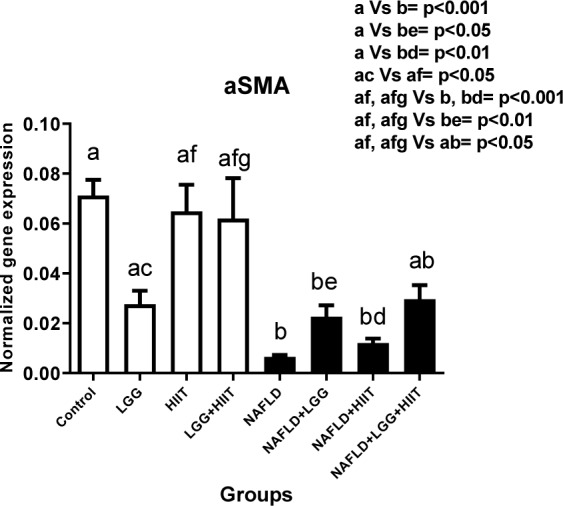
Liver tissue alpha-smooth muscle actin (α-SMA) mRNA expression in different groups of study. All values are the mean ± SD (n = 10 per group). Values with different superscripts are significantly different, p < 0.05. LGG: Probiotic *L. rhamnosus* GG, HIIT: High intensity interval training, NAFLD: Non-alcoholic fatty liver disease

As can be observed, all NAFLD groups except the combination therapy group (NAFLD+LGG+Exe) showed a significant decrease in *α-SMA* compared to the healthy control group (p < 0.05). Also, the decreasing changes in NAFLD+HIIT group compared to HIIT group (p < 0.001) and the decreasing changes in NAFLD+LGG group compared to LGG group (p < 0.01) were significant *in α-SMA*. However, in NAFLD groups, it was observed that different treatment modalities did not show a significant effect *on α-SMA* factor (p > 0.05) (Figure 5(Fig. 5)).

### MCP-1 mRNA

**Figure 6:**
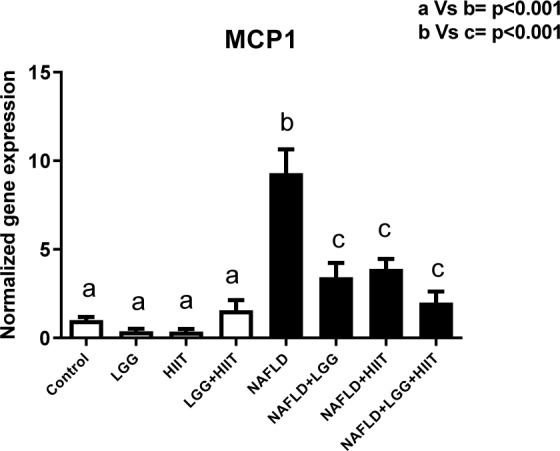
Liver tissue monocyte chemoattractant protein 1 (*mcp1*) mRNA expression in different groups of study. All values are the mean ± SD (n = 10 per group). Values with different superscripts are significantly different, p < 0.05. LGG: Probiotic *L. rhamnosus* GG, HIIT: High intensity interval training, NAFLD: Non-alcoholic fatty liver disease

Changes related to *MCP-1 *gene expression are shown in Figure 6(Fig. 6). As can be seen, changes in *MCP-1* gene are not significant in healthy groups (p> 0.05). However, NAFLD showed a significant increase in hepatic *MCP-1* compared to healthy groups with and without LGG and HIIT (p <0.001). Compared with NAFLD group, NAFLD+LGG, NAFLD +HIIT and NAFLD+LGG+HIIT groups showed a significant decrease in hepatic *MCP-1* (p <0.001).

### ACC mRNA

**Figure 7:**
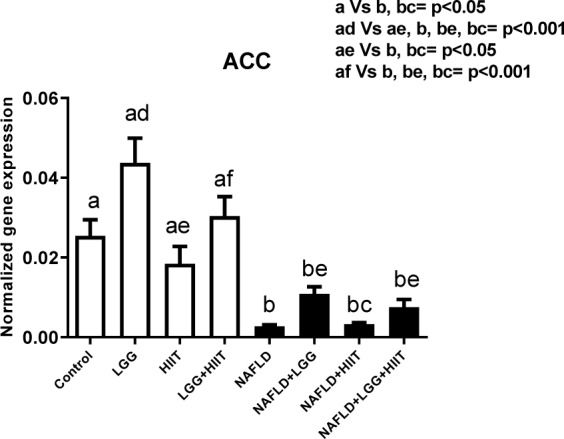
Liver tissue acetyl-CoA carboxylase (*ACC*) mRNA expression in different groups of study. All values are the mean ± SD (n = 10 per group). Values with different superscripts are significantly different, p < 0.05. LGG: Probiotic *L. rhamnosus* GG, HIIT: High intensity interval training, NAFLD: Non-alcoholic fatty liver disease

Hepatic *ACC* gene expression is shown in Figure 7(Fig. 7). In healthy groups, it was found that only changes in *ACC* gene expression between LGG and HIIT groups were significant (p < 0.001). However, compared to healthy control and HIIT groups, the NAFLD and NAFLD+HIIT groups showed a significant decrease in *ACC* (p <0.05). Compared to the LGG group, all NAFLD groups showed a significant decrease in liver *ACC*. Meanwhile, compared with LGG+HIIT group, all NAFLD groups showed a significant decrease in *ACC* gene expression in the liver (p < 0.001).

### APOC-3 mRNA

**Figure 8:**
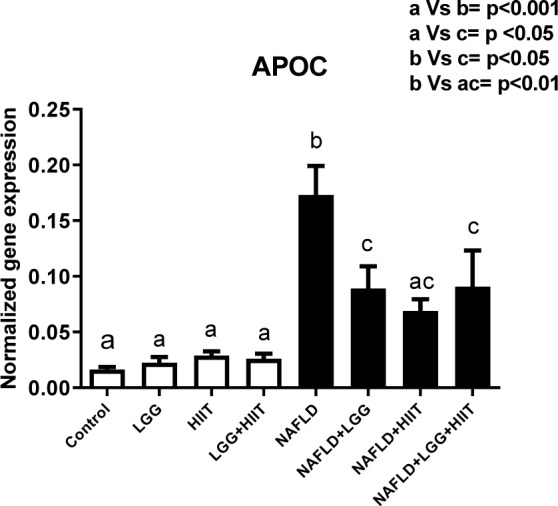
Liver tissue Apolipoprotein C (*apoc*) mRNA expression in different groups of study. All values are the mean ± SD (n = 10 per group). Values with different superscripts are significantly different, p < 0.05. LGG: Probiotic *L. Rhamnosus* GG, HIIT: High intensity interval training, NAFLD: Non-alcoholic fatty liver disease

The results showed that compared to the healthy groups, the *APOC-3* values in the NAFLD group significantly increased (p < 0.001) (Figure 8(Fig. 8)). Also, NAFLD+LGG and NAFLD+LGG+HIIT showed a significant increase in *APOC-3* mRNA compared to healthy groups (p < 0.05). Compared to NAFLD group, NAFLD+LGG (p < 0.05), NAFLD+HIIT (p < 0.01) and NAFLD+LGG +HIIT (p < 0.05) showed a significant decrease in *APOC-3* mRNA.

### LIPA and PNPLA3 mRNA 

**Figure 9:**
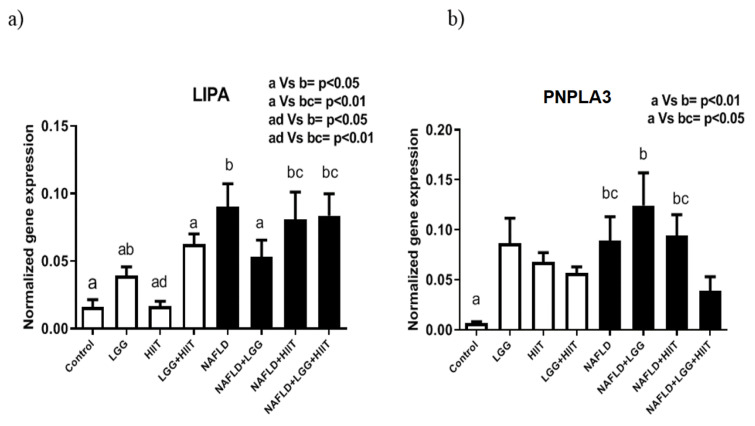
Liver tissue lysosomal acid lipase (*LIPA*) (a) and patatinlike phospholipase domain containing 3 (*pnPla3*), (b) mRNA expression in different groups of study. All values are the mean ± SD (n = 10 per group). Values with different superscripts are significantly different, p < 0.05. LGG: Probiotic *L. rhamnosus* GG, HIIT: High intensity interval training, NAFLD: Non-alcoholic fatty liver disease

The results showed that *LIPA* gene expression levels in NAFLD (p < 0.05), NAFLD +HIIT and NAFLD+LGG+HIIT (p < 0.01 for both) groups were significantly reduced compared to control and HIIT groups (Figure 9a(Fig. 9)).

Changes in *PNPLA3* gene expression are also shown in Figure 9b(Fig. 9). As can be seen, only the NAFLD (p < 0.05), NAFLD+LGG (p < 0.01) and NAFLD+HIIT (p < 0.05) groups showed a significant increase in *PNPLA3* gene expression compared to the control group. However, changes in this gene were not significant among healthy groups (Figure 9b(Fig. 9)).

### TGF-β and TNF-α mRNA

**Figure 10:**
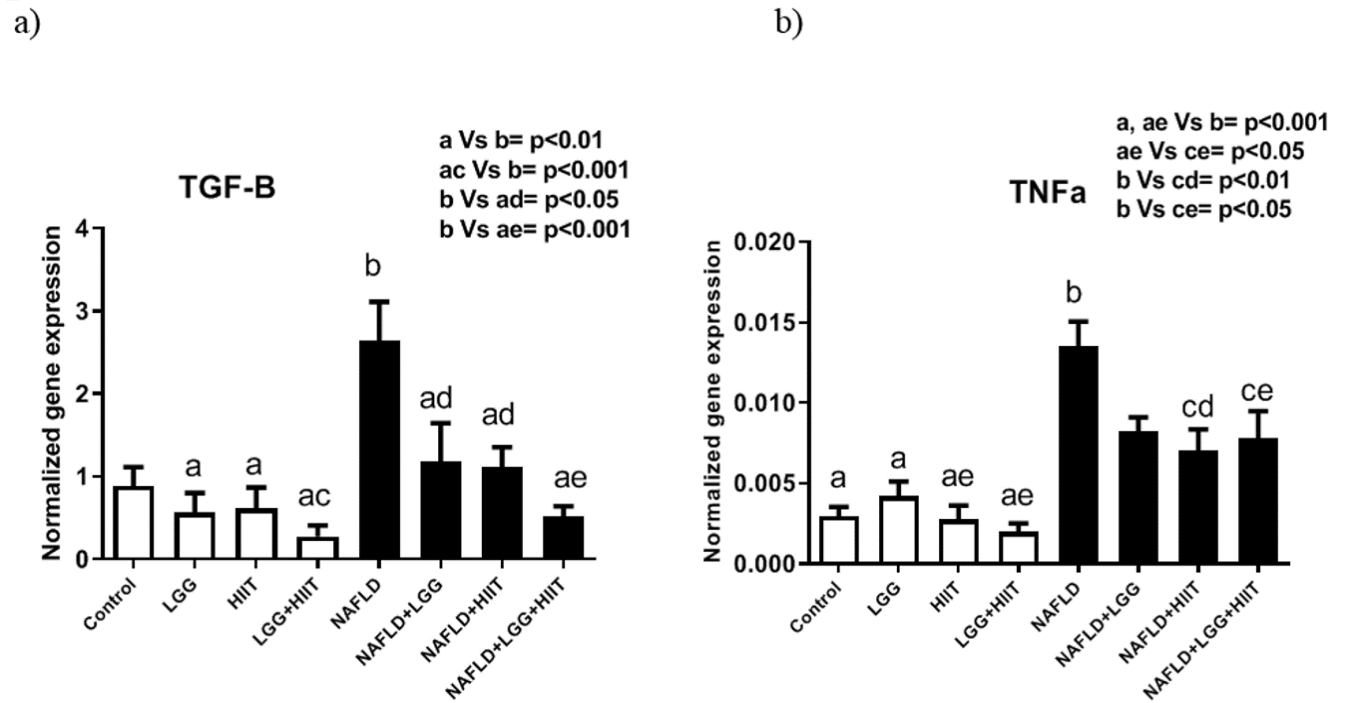
Liver transforming growth factor beta (*TGF-β*) (a) and tumor necrosis factor-α (*TNF*-α), (b) mRNA expression in different groups of study. All values are the mean ± SD (n = 10 per group). Values with different superscripts are significantly different, p < 0.05. LGG: Probiotic *L. Rhamnosus GG*, HIIT: High intensity interval training, NAFLD: Non-alcoholic fatty liver disease

*TGF*-*β* mRNA increased in NAFLD group, which was significant compared to LGG, HIIT (both p < 0.01) and LGG+HIIT (p < 0.01) groups (Figure 10a(Fig. 10)). However, NAFLD+LGG, NAFLD+HIIT (p < 0.05) and NAFLD+LGG+HIIT (p < 0.001) groups showed a significant decrease in *TGF-β *compared to NAFLD group (Figure 10a(Fig. 10)).

Changes in the inflammatory factor *TNF-α* are also shown in Figure 10b(Fig. 10). As can be seen, *TNF-α* mRNA levels in NAFLD group showed a significant increase compared to all healthy groups (p < 0.001). However, NAFLD+HIIT and NAFLD+LGG+HIIT groups showed a significant decrease in *TNF-α* mRNA compared to NAFLD group (p < 0.01 and p < 0.05, respectively).

## Discussion

Probiotics and exercise training can improve some liver functions, glucose, and lipid metabolism in patients with NAFLD. Thus, this study aimed to consider the ameliorative effects of interval exercise and *Lactobacillus rhamnosus GG* against tetracycline-induced fatty liver in rats.

Recently, widespread scientific attention has been paid to the use of probiotics due to its antioxidant and anti-inflammatory effects (especially in the control of intestinal microbiota) (Cristofori et al., 2021[15]; Wang et al., 2017[54]). Several studies have confirmed the antioxidant effects of *L. rhamnosus* GG and its effects on improving inflammation (Grompone et al., 2012[21]; Oh et al., 2018[39]). The results of the present study showed that in comparison with the fatty liver groups, the combination therapy group (NAFLD+LGG+HIIT) caused a significant decrease in *TIMP-1* mRNA compared to the NAFLD group. *TIMP-1*, also called fibroblast collagenase inhibitor, is a natural inhibitor of matrix metalloproteinases, a group of peptidases that are involved in the destruction of extracellular matrix (Visse and Nagase, 2003[51]). Consistent with the results of the present study, several studies have shown that *TIMP-1* in hepatic fibrosis increases in both mouse and human models and promotes the development of hepatic fibrosis. Various mechanisms are involved in the degradation function of *TIMP-1*. *TIMP-1* is generally thought to be secreted by satellite cells and Kupffer cells in the liver (Knittel et al., 1999[28]), but is also produced in hepatocytes under pathological conditions, such as carbon tetrachloride (CCl4)-induced hepatic fibrosis (Wang et al., 2011[53]). In one model of bile duct injury, it was shown that *TNF-α* may cause hepatic fibrosis by producing *TIMP-1* from liver satellite cells (Osawa et al., 2013[40]). In the present study, although the activity and secretion of liver satellite and Kupffer cells were not investigated, it seems that probiotics and HIIT exercises are effective in regulating *TIMP-1* secretion by both modulating the immune response and improving Kupffer homeostasis. Meanwhile, the down-regulation of *TGF-β* induced by exercise and probiotics (Figure 9a(Fig. 9)) may play a role in reducing *timp-*1 expression because *TIMP-1* transcription is increased by the Smad signaling pathway downstream of the *TGF-β* receptor (Liu et al., 2015[33]). *MMP-2* mRNA changes also revealed that NAFLD and NAFLD+HIIT groups showed a significant increase in hepatic *MMP-2* gene expression. However, compared to the NAFLD group, the NAFLD+LGG+HIIT group showed a significant decrease in *MMP-2*. Based on these results, it seems that exercise alone is not effective in controlling the expression of liver *MMP-2* gene in NAFLD conditions. Previous studies have indicated that strenuous exercise can lead to oxidative stress and inflammation (Bessa et al., 2016[6]; Li et al., 2016[31]). The exercise training of the present study is also intense; it seems that this high intensity exercise can also be harmful to the spread of inflammation. Since the combination therapy group (NAFLD+LGG+HIIT) significantly reduced the expression of *MMP-2* gene, it can be stated that supplementation with compounds containing antioxidant and anti-inflammatory properties can be effective in improving this stress response to strenuous exercise (Clifford et al., 2016[13]). In the present study, it seems that the use of this probiotic along with exercise can control the negative effects of intense exercise and be effective in reducing liver fibrosis.

Satellite cells in the liver under pathological stimulation can increase the production of fibrillar collagen (type I and type III collagen) (Safadi and Friedman, 2002[46]). Satellite cells also increase *α-SMA* production. Therefore, increased *α-SMA* expression is attributed to increased liver satellite cell activity. The results of the present study revealed that all NAFLD groups except the combination therapy group (NAFLD+LGG+Exe) had a significant decrease in *α-SMA* compared to the healthy control group. This indicates a decrease in satellite cell activity in the liver in NAFLD groups. There are conflicting studies on satellite liver cell activation, *α-SMA* secretion, and hepatic fibrosis. In most studies, satellite cell activation is associated with *α-SMA* and fibrosis. In the present study, satellite cells were not examined and changes in *α-SMA* were assessed only at the gene level. Also, the sensitivity of the assay between numerous studies can explain the lack of association between *α-SMA* and the severity of fibrosis in liver disease in the present investigation (Levy et al., 2002[30]).

Acetyl-CoA carboxylase (*ACC*) promotes the conversion of acetyl-CoA to malonyl-CoA and is involved in the metabolism of fatty acids (Tong, 2005[49]). The results of the present study showed that NAFLD groups had a significant decrease in *ACC* (type 1) compared to the probiotic group as well as LGG+HIIT. This result (decreased *ACC* in NAFLD group) indicates the degradation of fat metabolism in the liver by tetracycline. In mammals, there are two *ACC* isoforms, *ACC1* is cytosolic and participates in novo lipogenesis in liver and adipose tissue, while *ACC2* is in mitochondria and is involved in the down-regulation of β-oxidation in the heart and skeletal muscle (Abu-Elheiga et al., 2000[1]). TNF-α is one of the inflammatory factors that affects the liver *ACC*. It has been shown that *TNF-α* is involved in de novo lipid synthesis in the liver of mice. In the current study, *TNF-α* mRNA levels in NAFLD groups indicated a significant increase and it can therefore be stated that increasing this inflammatory factor can be effective in reducing the expression of *ACC* gene in liver tissue. However, although the therapeutic modalities of the study (HIIT and probiotics) increased *ACC* in healthy rats, HIIT and probiotics after fatty liver were not effective in controlling *TNF-α* and subsequent *ACC* regulation. Therefore, *TNF-α* may be indirectly involved in the ACCumulation of triglycerides in the liver, which may also be effective in inducing hepatic insulin resistance.

Chronic inflammation with mononuclear cell infiltration in the liver cell can also be common in patients with various liver injuries. In the present study, we measured the expression of the *MCP-1* gene. Consistent with other studies, our results showed that NAFLD significantly increased hepatic *MCP-1* compared to healthy groups. In comparison with NAFLD group, NAFLD+LGG, NAFLD+ HIIT and NAFLD+LGG+HIIT groups showed a significant decrease in hepatic *MCP-1*. Chemokines and chemokine receptors are significantly increased in liver disease (Coulon et al., 2012[14]). In obese conditions, it has been reported that the spread of inflammation due to adipose tissue causes high secretion of *MCP-1* from fat cells (Weisberg et al., 2006[55]). In this study, although inflammation due to obesity was not considered, the measurement of *TNF-α* indicates the spread of inflammation in liver tissue, which can also play a role in the regulation of hepatic *MCP-1*. Therefore, *TNF-α* may directly increase *MCP-1* production because *MCP-1* expression in the NAFLD group was in line with *TNF-α* expression. *TNF-α* can stimulate *MCP-1* gene transcription by activating the Akt/PKB pathway (Murao et al., 2000[38]). As a result, *TNF-α*-induced *MCP-1* expression may be an essential component in the progression of liver damage and cirrhosis. It seems that exercise and probiotic supplements can be effective in down-regulating *TNF-α* and *MCP-1* and improving liver disease. Also, *TNF-α* can affect some fibrosis factors. *TGF-β* families are among the genes associated with fibrosis. In chronic liver disease, *TGF-β* has been shown to activate satellite cells and fibroblasts, resulting in liver fibrosis (Lim et al., 2009[32]). Exercise and probiotic supplements have broad anti-inflammatory effects and can also be effective in down-regulating *TGF-β* by reducing inflammation (*TNF-α*).

In the study of other genes related to hepatic fat metabolism, the results of the present study revealed that compared to the healthy control group, the *apoc3* levels of NAFLD groups showed a significant increase (just NAFLD+HIIT), with the largest increase being relevant to NAFLD groups. Consistent with the results of this study, most studies have examined the genetic defects (mutations) in *apoc3* and have shown that defects in the function of this gene can increase blood flow triglycerides as well as liver damage such as fatty liver (Peter et al., 2012[41]). Therefore, the decrease in *apoc3* function or structure can improve blood lipid that affects liver metabolism. Our study shows that after NAFLD induction, the prescription of probiotics and HIIT or combined therapy significantly decrease *apoc3* mRNA in the liver. HIIT can improve weight by increasing fat burning. Also, probiotics can improve fat metabolism. Therefore, they can have an effect on *apoc3* and triglyceride metabolism. Hepatic *apoc3* expression has been shown to be physiologically inhibited by insulin, and its expression and secretion are increased in insulin-resistant states (Altomonte et al., 2004[3]). Exercise training is an agent that improves insulin sensitivity by increasing insulin receptors and glucose transporter in different tissues. Hence, the decrease of liver *apoc3* can relate to decreasing insulin resistance after HIIT.

Consistent with these changes, the NAFLD group also showed a significant increase in *PNLAP3* gene expression compared to the healthy control group. *PNLAP3* seems to be one of the genes that regulates fat ACCumulation in the liver, which can affect liver damage under mutation conditions. As with *apoc3*, a genetic defect in *PNPLA3* has been shown to be associated with NAFLD (Romeo et al., 2008[45]). In the present study, it was shown that although the induction of fatty liver caused a significant increase in *PNPLA3* in the liver, NAFLD+LGG+HIIT do not show a significant increase compared to the healthy control group. It seems that HIIT and probiotic therapy are prevention strategies that do not let *PNPLA3* mRNA increase in fatty liver (by improving fat metabolism). It is stated that loss of function in *PNPLA3* may associate with reduced release of retinol from lipid droplets and subsequent propensity to liver fibrosis (Pingitore et al., 2016[42]). In the present study, the improvement in *PNPLA3* function through HIIT and probiotics could be due to the improved release of retinol from lipid droplets, which is involved in the control of fat metabolism and fibrosis. However, in this study, changes in retinol were not examined. *LIPA* gene expression levels also increased significantly in NAFLD and NAFLD+HIIT and NAFLD+LGG+HIIT groups (compared to healthy control), while NAFLD+LGG group showed a significant decrease in hepatic *LIPA* compared to NAFLD group. Consistent with these changes, it has been shown that a defect in *LIPA* or lysosomal acid lipase deficiency (LAL-D) is part of the cause of undiagnosed liver disease, and a defect in this factor can lead to cirrhosis of the liver with primary dyslipidemia and atherosclerosis. In line with our results, it is stated that LAL-D leads to NAFLD (Himes et al., 2016[22]; Baratta et al., 2019[5]; Carotti et al., 2020[7], 2021[8]). In the present study, an increase in *LIPA* levels was observed in the NAFLD group. It seems that probiotics with antioxidant and anti-inflammatory properties have better effects on *LIPA* down-regulation in the liver tissue.

NAFLD is a common disease with a global prevalence of 25 % that can also be caused by poor diet and gastrointestinal damage (Powell et al., 2021[43]). To date, most research studies have considered the degradation of fat metabolism to be effective in the development of fatty liver and have suggested various treatment strategies for it. However, it is not yet clear whether the use of probiotics and exercise improves digestion of the fatty liver gene profile by improving digestion.

## Conclusion

According to the results of the present study, it seems that the use of HIIT training modality, which has a suitable intensity for fat control along with probiotic therapy, can well control the gene profile of factors related to fat metabolism and liver fibrosis, and have a therapeutic role. However, more studies are needed, especially on human trials.

## Declaration

### Author contribution

All authors have participated in drafting the manuscript. All authors reviewed the results and approved the final version of the manuscript. The authors declare that all data were generated in-house and that no paper mill was used. 

### Data availability

All the data is available upon a reasonable request. 

### Funding

This research received no specific grant from any funding agency.

### Declarations ethics

All animals were behaved and sacrificed following the National Institutes of Health guide for the care and use of laboratory animals (NIH Publications No. 8023, revised 1978).

### Competing interests

The authors have no conflicts of interest to declare.

## Figures and Tables

**Table 1:**
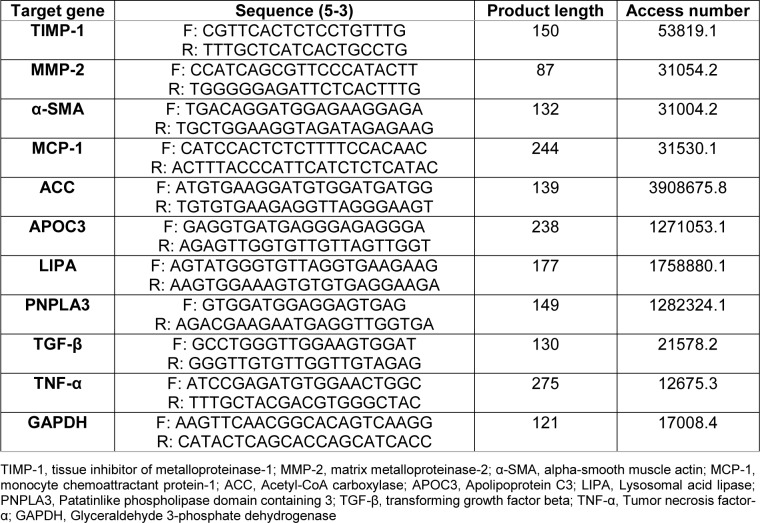
Sequences of oligonucleotides used as primers
